# Fulfillment of the premenstrual dysphoric disorder criteria confirmed using a self-rating questionnaire among Japanese women with depressive disorders

**DOI:** 10.1186/1751-0759-5-5

**Published:** 2011-05-02

**Authors:** Yoshiko Miyaoka, Yoshie Akimoto, Kayoko Ueda, Yuri Ujiie, Machiko Kametani, Yoko Uchiide, Toshiko Kamo

**Affiliations:** 1 Faculty of Letters, Atomi University, Saitama, Japan; 2 Institute of Women's Mental Health, Tokyo Women's Medical University, Tokyo, Japan; 3 Department of Psychiatry, Tokyo Women's Medical University, Tokyo, Japan

## Abstract

**Background:**

Some women with depressive disorders experience severe premenstrual symptoms. However, there have been few studies in which premenstrual symptoms in women suffering from depressive disorders were assessed. In this study, we aimed to investigate premenstrual symptoms in women with depressive disorders using the premenstrual dysphoric disorder (PMDD) scale.

**Methods:**

We administered questionnaires to 65 Japanese female outpatients who had been diagnosed with a major depressive disorder or dysthymic disorder and to 303 healthy women as control subjects. The questionnaire consisted of items on demographics and the PMDD scale, which was modified from the premenstrual symptoms screening tool (PSST) developed by Steiner et al. (*Arch Womens Ment Health *2003, **6**:203-209).

**Results:**

Twenty-eight women (43.1%) with depressive disorder fulfilled certain items of the PMDD scale. These women are considered to have coexisting PMDD and a depressive disorder, or to have premenstrual exacerbation (PME) of a depressive disorder. On the other hand, 18 women (5.9%) in the control group were diagnosed as having PMDD. The depressive disorder group who fulfilled the PMDD criteria had more knowledge of the term premenstrual syndrome (PMS) and took more actions to attenuate premenstrual symptoms than the control group with PMDD.

**Conclusions:**

Our findings demonstrated that the occurrence of severe premenstrual symptoms is much higher in women with depressive disorders than in healthy subjects. This is partially due to this group containing women with PME, but mainly due to it containing women with PMDD. The higher percentage of PMDD suggests similarity between PMDD and other depressive disorders. Furthermore, educating healthy Japanese women and women with depressive disorders about premenstrual symptoms and evidence-based treatment for them is necessary.

## Introduction

Approximately 80% of all women of reproductive age experience physical and/or psychological changes in the late luteal phase; i.e., the premenstrual period [1]. Physical changes include breast swelling, fatigue, headache, and weight gain. Psychological changes include depressive mood, irritability, and tension. These changes remit after the onset of menstruation and are commonly called premenstrual syndrome (PMS). However, PMS has a broad concept and varies in severity. In the International Classification of Diseases (10^th ^revision; ICD-10), PMS was listed as "premenstrual tension" under "Diseases of the Genitourinary System" [2].

Although many women complain of mild symptoms, approximately 5% of women suffer from severe symptoms that lead to a reduction in quality of life [1]. In the field of psychiatry, the diagnostic and statistical manual of mental disorders, 3^rd ^edition, revised (DSM-III-R) proposed a new diagnosis "late luteal phase dysphoric disorder (LLPDD)" in an appendix for further research [3]. LLPDD is a severe variant of PMS and a dysphoric disorder that interferes with social and occupational functioning during the premenstrual period. Physical symptoms are not required for the diagnosis of LLPDD. In the diagnostic and statistical manual of mental disorders, 4^th ^edition (DSM-IV) [4], a new diagnosis, "premenstrual dysphoric disorder (PMDD)" is listed as a criterion for further study instead of LLPDD. The PMDD diagnosis is also based on the assumption that the condition with the premenstrual symptoms is mainly psychiatric and should be separated from a condition with merely somatic complaints. Physical complaints are not mandatory symptoms to meet the criteria for PMDD or LLPDD.

The outline of the criteria for PMDD is shown in Table 1. Section A shows a list of symptoms. At least one of the symptoms, 1, 2, 3, or 4, is required for the diagnosis. Symptoms 1, 2, 3, and 4 are related to moods in depression. Section B shows a list of symptoms associated with disturbance in ordinary life and relationships with others. In section C, it is stated that an exacerbation of another psychiatric disorder should be ruled out. In section D, it is suggested that the diagnosis should be confirmed prospectively within at least 2 months. However, a provisional diagnosis that is not confirmed prospectively is allowed.

**Table 1:** Outline of PMDD criteria in DSM-IV [4]

A.	In the menstruation cycles during the past year, the following symptoms were present during the week prior to menstruation, began to remit within a few days after the onset of menstruation, and disappeared in the week postmenstruation.							
	Five symptoms, including one of (1), (2), (3), or (4) are necessary for diagnosis							
								
1	Depressive mood/feelings of hopelessness							
2	Anxiety/tension							
3	Tearful/feeling suddenly sad							
4	Anger/irritability/increased interpersonal conflicts							
5	Decreased interest in work, school, or hobbies							
6	Difficulty in concentrating							
7	Easily fatigued/lethargy							
8	Overeating/specific food cravings							
9	Hypersomnia/Insomnia							
10	Feeling out of control							
11	Physical symptoms such as breast tenderness or weight gain							
								
B.	The disturbance interferes with work, school, usual social activities, or relationships with others.							
								
C.	The disturbance is not an exacerbation of another disorder; such as another depressive disorder or panic disorder.							
								
D.	Criteria must be confirmed using a prospective daily rating scale during at least two consecutive symptomatic cycles. (The diagnosis may be made provisionally prior to this confirmation.)							

Because PMDD is a proposed diagnosis in DSM-IV, PMDD is diagnosed as a "depressive disorder not otherwise specified". This indicates that PMDD is equivalent to other depressive disorders in some way. Many studies have found a correlation between premenstrual symptoms and depression. For example, women with PMDD or PMS have a greater history of depression [5,6]. Lane and Francis [7] estimated the percentage of women with a lifetime history of affective disorders who have premenstrual changes at roughly 60%. Moreover, women with premenstrual symptoms demonstrated a more severe depression than the control group [8-10].

In most studies, premenstrual symptoms in healthy subjects have been investigated. However, studies in which premenstrual symptoms were assessed in women suffering from depressive disorders are few [5,11,12]. One reason for this is the difficulty in defining premenstrual symptoms. If a woman with a depressive disorder experiences premenstrual symptoms, these symptoms may coexist with PMS or occur as premenstrual exacerbation (PME) of the depressive disorder. It is somewhat difficult to differentiate them. In this study, we examined the percentage of women with depressive disorders who fulfilled PMDD criteria using a self-rating questionnaire; these women may have included those with PME, as well as those with PMDD. We compared the characteristics of these women with healthy subjects with PMDD.

## Methods

### Subjects

This study was conducted from June to December in 2008 at a mental care clinic in the Institute of Women's Mental Health, Tokyo Women's Medical University. The ethics committee of Tokyo Women's Medical University approved the conduct of this study.

Sixty-five female outpatients who had been diagnosed as having a major depressive disorder or dysthymic disorder on the basis of DSM-IVcriteria [4] and whose ages ranged from 20 to 45 years old were recruited. They gave their written informed consent and completed a questionnaire. Through schools and offices, the questionnaire was also distributed to 435 students, employed women, working mothers, and housewives as a control group. Their ages ranged from 20 to 45 years. Three hundred twenty two women responded. Nineteen(5.9%)of those who reported they were suffering from depressive disorders or had a history of a depressive disorder were excluded. The remaining subjects (303) were analyzed.

### Procedure

The questionnaire consisted of items on demographics and the PMDD scale.

#### 1. Demographics

Demographic information such as marital status, children, and smoking was obtained. We also asked information about the respondents' menstruation: regularity of cycles, physical symptoms during menstruation, knowledge of PMS, and taking actions to attenuate its symptoms.

#### 2. PMDD scale

We developed the PMDD scale [13] based on DSM-IVcriteria [4], which was modified from the premenstrual symptoms screening tool (PSST) developed by Steiner et al. [14]. The scale was found to have high reliability and validity [13]. Respondents were first asked whether they had any symptoms listed in the questionnaire that begin to appear one or two weeks prior to the start of menstruation and disappear 2 or 3 days after menstruation in most menstrual cycles during the past year.

There are two parts to the PMDD scale. In the first part, symptoms that were quoted from section A of the PMDD criteria in DSM-IV [4] were listed. There are 11 symptoms listed in DSM-IV (Table 1). We formulated two questions to distinguish between hypersomnia and insomnia in section A, criterion 9. Therefore, the PMDD scale consisted of 12 symptoms in section A. If one answers "yes" (mild to severe) for at least one symptom, she needed to answer the section B criteria in DSM-IV. It consisted of five correlations about menstruation interfering with activities or relationships.

As a result of a factor analysis, we found that there were three factors in the scale, namely, "fatigue and/or physical symptoms", "depressive moods", and "dysfunctional relationships and/or anger" [13].

We diagnosed a subject as having PMDD if she answered "severe" for at least one of the following items in section A: depressive mood, anxiety, tearfulness, and anger; answered "moderate" or "severe" for at least four items in section A; and also answered "severe" for at least one of the items in section B regarding the interference with activities and relationships.

### Statistical analysis

Statistical analysis was conducted using SPSS version 16.0. The Student t-test and χ^2 ^test were used to compare the variables between the depressive disorder and control groups. Significance tests were two-tailed.

## Results

We compared 65 patients who were diagnosed with major depressive disorder or dysthymic disorder and 303 control women in the general population who were not suffering from depressive disorder and did not have a history of it. Their ages ranged from 20 to 45 years. We compared the average total score of the PMDD scale between younger subjects (≦29 years old) and older subjects (≧30 years old) using the Student t-test. In the depressive disorder group, there was no significant difference between the younger sub group and the older sub group (younger, 41.9, vs. older, 45.9; p > .05). Similarly, in the control group, there was no significant difference between the younger group and the older group (younger, 31.6 vs. older, 29.5; p > .05).

Table 2 shows the characteristics of the two groups. The average age of the depressive disorder group was significantly higher than that of the control group. However, the average ages of the two groups were in the early 30's; 34.8 years and 31.9 years old. The two groups did not significantly differ in employment status, regularity of the menstrual cycle, and incidence of abdominal pain and/or lumbago during menstruation. Statistically significant differences were observed for marital status, parity, smoking, and knowledge of the term PMS.

**Table 2:** Characteristics of patients with depressive disorders and control subjects

Variable	Depressive disorder group	Control group	P
Number	65	303	
Average age (SD)	34.8 (7.5)	31.9 (7.9)	0.007
Employed			
Yes	33	136	n.s.
No	32	166	
Married			
Yes	17	147	0.001
No	47	156	
Have children			
Yes	16	117	0.033
No	49	186	
Smoke cigarettes			
Yes	18	33	0.000
No	47	267	
Regular menstrual cycle			
Yes	55	251	n.s.
No	9	52	
Abdominal pain/lumbago in menstruation			
Yes	59	263	n.s.
No	6	38	
Know the term PMS			
Yes	56	213	0.009
No	9	90	

SD: standard deviation

n.s.: not significant

PMS: premenstrual syndrome

Student t-test was used for the average age.

χ^2 ^test was used for other variables.

Table 3 shows the characteristics of the two groups of women. The first group consisted of 28 women (43.1%) with variables for depressive disorders fulfilling the criteria of PMDD determined using the PMDD scale. The second group consisted of 18 women (5.9%) in the control group with variables also fulfilling the criteria of PMDD determined using the PMDD scale. The percentage of satisfaction of the criteria of PMDD in the depressive disorder group was significantly higher than that in the control group. The two groups of women who fulfilled the criteria did not significantly differ in employment or marital status, parity, smoking, or incidence of abdominal pain and/or lumbago during menstruation. There were significant differences between the groups in regard to the regularity of the menstrual cycle, knowledge of the term PMS, and taking actions to attenuate premenstrual symptoms.

**Table 3:** Characteristics of women fulfilling PMDD criteria in depressive disorder and control groups

Variable	Fulfillment of PMDD criteria in depressive disorder group	Fulfillment of PMDD criteria in control group	P
Fulfillment of PMDD criteria	28 (43.1%)	18 (5.9%)	0.000
Average age (SD)	34.9 (7.2)	30.7 (7.7)	n.s.
Employed			
Yes	14	7	n.s.
No	14	11	
Married			
Yes	7	7	n.s.
No	21	11	
Have children			
Yes	7	8	n.s.
No	21	10	
Smoke cigarettes			
Yes	9	1	n.s.
No	19	17	
Regular menstrual cycle			
Yes	26	13	0.020
No	1	5	
Abdominal pain/lumbago in menstruation			
Yes	25	15	n.s.
No	3	3	
Know the term PMS			
Yes	25	11	0.024
No	3	7	
Taking action to attenuate symptoms			
Yes	22	9	0.044
No	6	9	

SD: standard deviation

n.s.: not significant

PMDD: premenstrual dysphoric disorder

PMS: premenstrual syndrome

Student t-test was used in the average age.

χ^2 ^test was used for other variables.

Table 4 shows the PMDD scale scores of the women who fulfilled the PMDD criteria in the depressive disorder and control groups. The total PMDD score of the women who fulfilled the PMDD criteria in the depressive disorder group was higher than that of those in the control group. There are three subscales. The depression mood score of those who fulfilled the PMDD criteria in the depressive disorder group was significantly higher than that in the control group. There were no significant differences in the scores for the items on fatigue/physical symptoms and the dysfunctional relationship/anger.

**Table 4:** PMDD scale scores of women fulfilling PMDD criteria in depressive disorder and control groups

	Fulfillment of PMDD criteria in depressive disorder group		Fulfillment of PMDD criteria in control group		P
	mean	SD	mean	SD	
Score of subscales					
Fatigue/physical symptoms	24.3	3.2	23.0	3.3	n.s.
Depression mood	13.4	2.9	9.4	3.5	0.000
Dysfunctional relationship/anger	17.4	3	16.2	2.3	n.s.
Total score	55.1	6.1	48.6	7.3	0.002

SD: standard deviation

n.s.: not significant

PMDD: premenstrual dysphoric disorder

Student t-test was used.

Table 5 shows the item scores for the women who fulfilled the PMDD criteria in the depressive disorder and control groups. The scores for depressive mood, anxiety, tearfulness, decreased interests, and interference with housework were significantly higher in the depressive disorder group. There were no significant differences in the scores for other items between the two groups.

**Table 5:** Item scores of women fulfilling PMDD criteria in depressive disorder and control groups

		Fulfillment of PMDD criteria in depressive disorder group		Fulfillment of PMDD criteria in control group		P
	Number	28		18		
		Mean	SD	Mean	SD	
A	Symptoms					
1	Depressive mood/feelings of hopelessness	3.39	0.92	2.67	1.09	0.019
2	Anxiety/tension	3.39	0.79	2.56	1.15	0.011
3	Tearful/feeling suddenly sad	3.54	0.74	2.00	1.19	0.000
4	Anger/irritability/increased interpersonal conflicts	3.82	0.67	3.94	0.24	n.s.
5	Decreased interest in work, school, or hobbies	3.07	1.12	2.17	1.04	0.009
6	Difficulty in concentrating	3.25	1.01	3.33	0.59	n.s.
7	Easily fatigued/lethargy	3.75	0.65	3.72	0.58	n.s.
8	Overeating/specific food cravings	3.32	1.16	2.83	1.25	n.s.
9	Hypersomnia	3.32	1.02	3.44	0.98	n.s.
10	Insomnia	1.50	1.11	1.33	0.59	n.s.
11	Feeling out of control	3.75	0.70	3.33	1.03	n.s.
12	Physical symptoms†	3.93	0.26	3.78	0.55	n.s.
						
B	Interfere with activities or relationships					
1	Interfere with efficiency at work or school	3.18	1.25	3.00	1.14	n.s.
2	Interfere with housework	3.57	0.84	2.89	1.13	0.023
3	Interfere with relationships with coworkers	2.36	1.50	1.78	1.17	n.s.
4	Interfere with relationships with family	3.32	1.09	3.39	0.78	n.s.
5	Interfere with relationships with friends and acquaintances	2.61	1.07	2.39	1.20	n.s.

SD: standard deviation

n.s.: not significant

PMDD: premenstrual dysphoric disorder

†Symptoms such as breast tenderness or swelling, abdominal pain, headaches, joint or muscle pain, sensation of bloating, weight gain, or constipation.

Student t-test was used.

## Discussion

### The percentages of women with depressive disorders who fulfilled PMDD criteria

Before focusing on the main issue of this section, we would firstly like to review some studies of the prevalence of PMDD among healthy women. Endicott [15] pointed out that only about 3% to 8% of women have sufficiently severe symptoms to meet the DSM-IV criteria for PMDD, whereas many women experience some premenstrual symptoms. In studies using diagnostic structured interviews administered in community samples, Wittchen et al. [16] observed that the prevalence of PMDD was 5.8% and Potter et al. [17] identified 12.2% of women having PMS and 4.1% having severe PMS, which may have severe premenstrual symptoms similar to PMDD symptoms. There were more studies using questionnaires for the assessment of PMDD. Wallenstein et al. [18] administered a questionnaire to women who were 18-45 years old and were not being treated for depression-related disorders. They identified 6.0% of these women as being at risk of PMDD. Cohen et al. [19] diagnosed 6.4% of older women (36-44 years old) as having PMDD using self-rating daily records across a menstrual cycle. Otsubo and Owashi [20] reported that 4.2% of women were identified as having PMDD using a questionnaire based on the criteria of DSM-IV. Ito et al. [21] observed that 8.6% of healthy women appeared to have PMDD, as determined using a questionnaire. Steiner et al. [14] determined the prevalence of PMDD to be 4.1% using the PSST. In Japan, Takeda et al. [22] developed a scale that was modified from the PSST developed by Steiner et al. [14] and observed a 1.2% prevalence of PMDD in Japan. It was the lowest among those previously reported. The PMDD scale that we developed was also modified from the PSST. In our previous study [13], the percentage of Japanese women in a healthy group who fulfilled the PMDD criteria was 5.9%. Our finding was similar to the prevalence determined using the PSST developed by Steiner et al. and those in many other studies.

Regarding the relationship between PMDD and depression, we found that 43.1% of the women with depressive disorders fulfilled the PMDD criteria determined using the PMDD scale. Many researchers have pointed out high rates of a history of or comorbidity of other depressive disorders in women with PMDD or PMS [5,6,11,12]. Soares et al. [6] found that the prevalence of PMDD was 6.3% and that over one half of the women (55.6%) had a history of a depressive disorder. Ito and Matsubara [12] observed that 31% of women with depressive disorders were identified as having PMDD using a questionnaire. Halbreich and Endicott [5] evaluated current mental diagnoses using the Research Diagnostic Criteria (RDC) and premenstrual dysphoric changes using the Premenstrual Assessment Form (PAF) in 170 women. Eighty-four percent of women who met the criteria of major depressive disorder also had depressive mood changes during their premenstrual period, whereas only 9% of women who were not mentally ill experienced these mood changes. In our current and previous reports [13], 43.1% of the women with depressive disorders fulfilled PMDD criteria, whereas 5.9% of the women without depressive disorders did. Their findings support ours.

The diagnosis of PMDD is categorized as "depressive disorder not otherwise specified". This categorization indicates that PMDD is closely related to depression in symptomatology and characteristics. It suggests that premenstrual changes, which include mild symptoms, are also associated with mood changes. The issue of whether women with PMS exhibit a greater severity of depression throughout an entire menstrual cycle than those without PMS is controversial [7]. In a report of Morse et al. [8], women with PMS scored a significantly greater severity of depression determined using the Beck depression inventory (BDI) than control subjects who did not complain of any premenstrual symptom in the premenstrual phase. However, there was no difference between the groups in the severity of depression in the follicular phase. On the other hand some studies showed that women experiencing premenstrual changes demonstrate a greater severity of depression in not only the premenstrual phase but also in the remaining phases of the menstrual cycle [23,24].

### Possibility of premenstrual symptoms being PMDD or PME (premenstrual exacerbation)

The questionnaire cannot rule out the possibility of an exacerbation of symptoms of depressive disorders. Therefore, when a woman with a depressive disorder fulfills the PMDD criteria determined using the questionnaire, her premenstrual symptoms cannot be clearly defined as being coexistent with PMDD or PME. Previous studies indicated a high prevalence of PME in women with a depressive disorder that ranged from 25% to 80%. Kornstein et al. [25] showed that 64% of women with a major depressive disorder reported premenstrual worsening. Hsiao et al. [11] used structured interviews and reported that the frequency of PMS was 80% in patients with depressive disorders. This 80% included cases of premenstrual exacerbation. The frequency of premenstrual exacerbation was also high (52%). Depressive patients who fulfill the PMDD criteria may include those whose symptoms are merely an exacerbation of symptoms of depressive disorders when a self-rating scale is used. This may be one of the reasons why the occurrence of premenstrual symptoms in the depressive disorder group was higher than that in the control group. In the criteria of PMDD in DSM-IV, an exacerbation of symptoms of other psychiatric disorders should be ruled out. However, it is difficult to distinguish PMDD from an exacerbation of symptoms of other psychiatric disorders because the PMDD scale is self-rating. Although patients with premenstrual worsening of depressive disorders could not be ruled out using the scale in the present study, the results showed that the percentage of the women with depressive disorders who experienced severe premenstrual symptoms was higher than that of healthy subjects. It suggests similarity between PMDD and other depressive disorders.

### Characteristics of women with depressive disorders who fulfilled PMDD criteria

We compared the PMDD symptoms listed in the questionnaire between the patients with depressive disorder and the control group in terms of the total score and depression mood subscale score (Table 4). The total score and depression subscale score were higher in the depressive disorder group. The depression mood subscale consists of five items; 'depressive mood/feeling of hopelessness', 'anxiety/tension', 'tearful/feeling suddenly sad', and 'decreased interest in work, school or hobbies'. The mean scores of all items of the depression mood subscale among the depressive patients who fulfilled the PMDD criteria were significantly higher than those of the control subjects who were diagnosed as having PMDD using the PMDD scale. In most other items, the mean score was not significantly different between the group with depressive disorders and the control group. However, the group with depressive disorders scored significantly higher in the item for interference with housework, which was included in the dysfunctional relationship/anger subscale (Table 5). The percentage of married women who met the PMDD criteria was not significantly different between the two groups (Table 3). Therefore, the result was not due to marital status. It is suggested that depressive women who fulfilled the PMDD criteria feel the burden of housework in the premenstrual period. We should pay attention to interference with housework as well as deterioration of depressive moods in the premenstrual period in depressive patients.

We compared the demographic characteristics between the patients with depressive disorders who fulfilled the PMDD criteria and control subjects who also fulfilled them. Soares et al. [6] observed that women with PMDD who also had a history of a depressive disorder were more likely to smoke, were less educated, and more commonly married than the women with PMDD without a history of it. However, we found no significant difference in smoking or marital status between the depressive disorder group who fulfilled the PMDD criteria and the control group who fulfilled them (Table 3). The present study revealed that the patients in the depressive disorder group who fulfilled the PMDD criteria were more knowledgeable about the term "PMS" than the control subjects who were diagnosed as having PMDD. Some patients might have known about PMS from being asked by their doctors. The depressive patients with PMDD or PME also took more actions to attenuate premenstrual symptoms than the control subjects with PMDD. The actions taken include taking medicine prescribed by doctors. Further education on premenstrual symptoms seems to be required for healthy women because of their poor knowledge of PMS and women with depressive disorder because of the high percentage of such women with severe premenstrual symptoms.

### Limitations

There were several methodological limitations of this study. Firstly, the sample size of patients was small and they are only outpatients who had attended only one clinic. Therefore, they could not represent all female patients with depressive disorders. Secondly, as we mentioned earlier, the questionnaire cannot rule out the possibility of exacerbation of symptoms of depressive disorders. There is no certainty as to whether the patients who fulfilled PMDD criteria had PMDD or PME. Thirdly, the subjects were identified using a self-rating questionnaire and diagnostic interviews were not conducted. As a result, the determinations of PMDD and PME were not conducted precisely. Lastly, it is recommended that a diagnosis of PMDD should be confirmed after an observation lasting at least 2 months. However, the questionnaire was administered only once, not prospectively. Further study to investigate PMDD or PME among women with depressive disorders is needed

## Competing interests

The authors declare that they have no competing interests.

## Authors' contributions

YM conceived the study. YM, YA, KU, and TK designed the study. YM and YA analyzed and interpreted the data. YUj, MK, and YUc participated in the design and coordination of the study. All authors read and approved the final manuscript.
